# Supplementary material for: Assessment of the quality attributes and oxidative stability of fish balls with the addition of fig powder during frozen storage
Source: PLoS One. 2025 Dec 1;20(12):e0335408. doi: 10.1371/journal.pone.0335408 (PMC12668626; doi:10.1371/journal.pone.0335408)
Supplement: Attachment — Submitted filename: Response to Reviewers.docx [file pone.0335408.s003.docx]

**Rebuttal Letter**

Dear Editor,

**Plos One**

Thank you so much for sending our manuscript to an anonymous reviewer. We have tried to address all the comments raised by the reviewers. Please see our following responses.

All revisions are yellow highlighted under track change. We have also added a clean version of the revised manuscript. If you need any more explanations, please inform me.

Ashrafuzzaman Zahid

Corresponding author

**Reviewer #1:**

**Comment 1:** The manuscript presents a technically sound piece of research with adequate experimental design and relevant data that support the conclusions. The study is well-structured, and the findings regarding the effect of BHT, AA, and fig powder on the physicochemical properties of fish balls are clearly presented.

**Response:** Thank you for the encouraging feedback. We are glad that our design, data, and findings on BHT, AA, and fig powder in fish balls were clear.

**Comment 2:** The statistical analysis appears to have been conducted appropriately and rigorously, supporting the interpretation of the results.

**Response:** Thank you for recognizing that the statistical analysis was conducted properly and supports our results.

**Comment 3:** The data underlying the study seem to be sufficiently available to support transparency and reproducibility. This strengthens the reliability of the manuscript.

Response: Thank you for noting the availability of our data. We are glad it supports transparency, reproducibility, and reliability of the manuscript.

**Comment 4:** The manuscript is generally well-written and presented in intelligible English. However, there are a few minor grammatical and typographical errors that should be carefully revised during the final editing stage to improve clarity and readability.

**Response:** We appreciate the reviewer’s comment. We have carefully revised the manuscript to correct the minor grammatical and typographical errors, improving overall clarity and readability. Please see the revised manuscript.

**Comment 5:** Overall, the study provides valuable insights into the potential of fig powder as a natural antioxidant alternative to synthetic compounds. With minor language polishing, the manuscript would be suitable for publication.

**Response:** We thank the reviewer for recognizing the value of our study. We have carefully polished the language throughout the manuscript to ensure it is clear and ready for publication. Please see the revised manuscript.

**Reviewer #2:**

The manuscript treats an important issue regarding the food safety and consumers' health that is the replacement of the synthetic antioxidants with natural ones. The overall quality of the paper is high; however, I have some minor remarks.

**Comment 1:** The Introduction provides the necessary strong background to justify the research. However, the authors provide information about the amount of fishballs produced in 2015. This is old data, please, provide more recent.

**Response:** We thank the reviewer for this suggestion. We have updated the manuscript with more recent data on fish ball production to provide a current and relevant background. Please see the revised manuscript line number 34-37.

**Comment 2:** How many fishballs does the batch contain for analysis? Please describe in more details. This is important for the statistical evaluation.

**Response:** We thank the reviewer for this comment. Each batch for analysis contained 48 fish balls. The statistical analysis was conducted based on four treatments × three replications × three storage times, as described in the manuscript. Please see the revised manuscript in line number 103 and lines 165.

**Comment 3:** How have the authors decided to use 1% fig powder? Why haven't they considered various concentrations of this powder?

**Response:** We thank the reviewer for this important comment. The 1% fig powder concentration was selected based on preliminary trials and literature reports indicating its effective antioxidant impact without adversely affecting the sensory properties of fish balls.

**Comment 4:** for each of the analysis, please provide the number of replicates and also the calculations for TBARS and DPPH.

**Response:** We have added the replication number (four treatments × three replications × three storage times) in the statistical analysis please see the line number 165. We have also added the calculations for DPPH. Please see the line numbers 153 in revised manuscript. Please see the line 132 for TBARS.

**Comment 5:** Although the one-way ANOVA is generally ok, here tow way ANOVA might be applied as well to assess the treatment, storage time and their interaction.

Response: We appreciate the reviewer’s suggestion regarding the use of two-way ANOVA. We have done one way ANOVA two times separately. Firstly, we have done it across the treatments and secondly across the storage time. It shows the similar results to two-way ANOVA. In our upcoming analysis we will do the two-way ANOVA according to your suggestion.

**Editor' s Comments:**

**Comment 1:** Authors need to improve the discussion and future recommendations in the MS.

**Response:** Thank you for your comment. We have revised and expanded the discussion section and added more detailed future recommendations to strengthen the manuscript. Please see the revised manuscript.
